# Human Parietal Cortex Structure Predicts Individual Differences in Perceptual Rivalry

**DOI:** 10.1016/j.cub.2010.07.027

**Published:** 2010-09-28

**Authors:** Ryota Kanai, Bahador Bahrami, Geraint Rees

**Affiliations:** 1 UCL Institute of Cognitive Neuroscience, University College London, 17 Queen Square, London WC1N 3AR, UK; 2 Wellcome Trust Centre for Neuroimaging, University College London, 12 Queen Square, London WC1N 3BG, UK; 3 Interacting Minds Project, Institute of Anthropology, Archaeology and Linguistics, Aarhus University and Centre of Functionally Integrative Neuroscience, Aarhus University Hospital, Norrebrogade 44, Building 10G, 8000 Aarhus C, Denmark

**Keywords:** SYSNEURO

## Abstract

When visual input has conflicting interpretations, conscious perception can alternate spontaneously between competing interpretations [1]. There is a large amount of unexplained variability between individuals in the rate of such spontaneous alternations in perception [2–5]. We hypothesized that variability in perceptual rivalry might be reflected in individual differences in brain structure, because brain structure can exhibit systematic relationships with an individual's cognitive experiences and skills [6–9]. To test this notion, we examined in a large group of individuals how cortical thickness, local gray-matter density, and local white-matter integrity correlate with individuals' alternation rate for a bistable, rotating structure-from-motion stimulus [10]. All of these macroscopic measures of brain structure consistently revealed that the structure of bilateral superior parietal lobes (SPL) could account for interindividual variability in perceptual alternation rate. Furthermore, we examined whether the bilateral SPL regions play a causal role in the rate of perceptual alternations by using transcranial magnetic stimulation (TMS) and found that transient disruption of these areas indeed decreases the rate of perceptual alternations. These findings demonstrate a direct relationship between structure of SPL and individuals' perceptual switch rate.

## Graphical abstract

**Graphical Abstract:**

**Figure:**
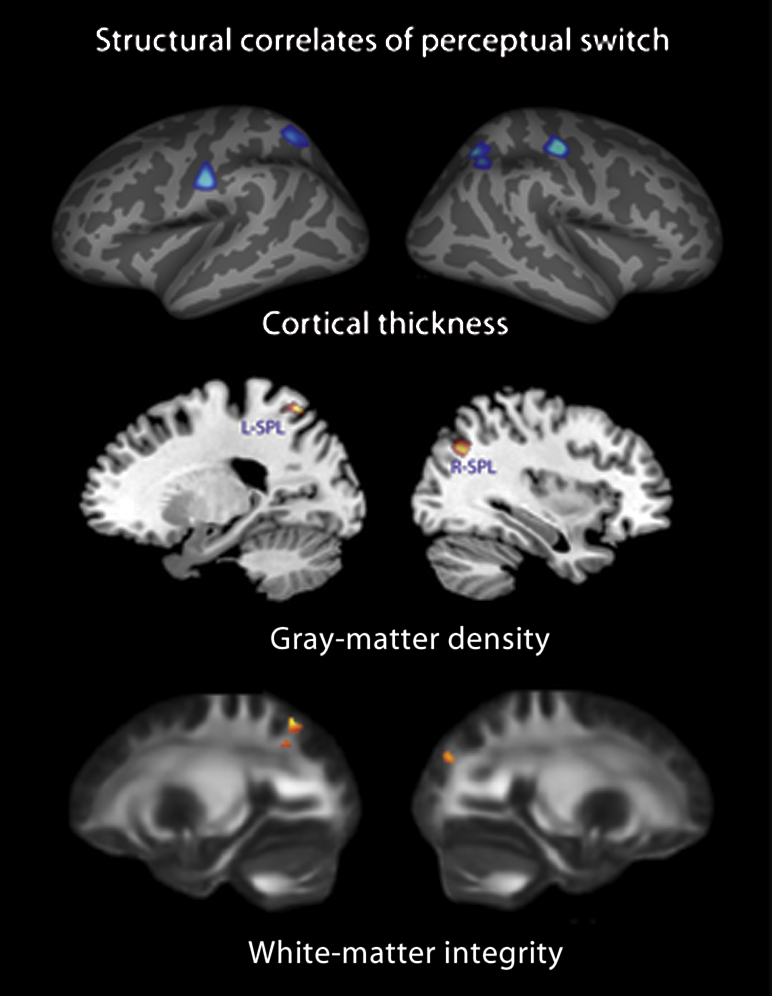

**Highlights:**

► Structure of superior parietal lobe (SPL) predicts switch rate in perceptual rivalry ► White-matter integrity in SPL correlates with individuals' switch rate ► Deactivation of SPL with transcranial magnetic stimulation slows perceptual rivalry

## Results

**Figure 1:**
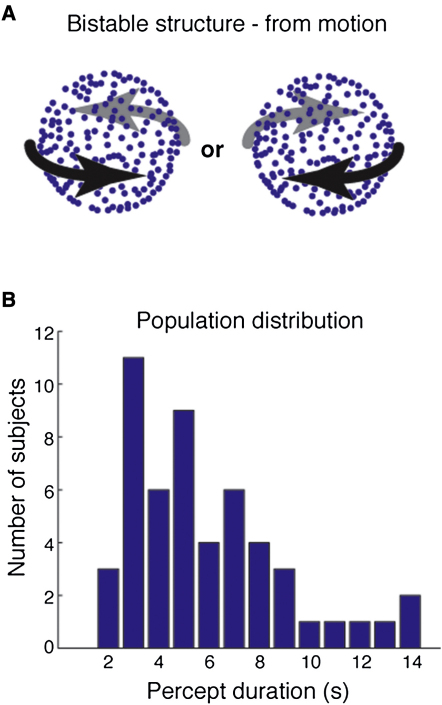
Illustration of Bistable Structure-from-Motion Stimulus and Individual Differences in Percept Duration (A) Sinusoidally oscillating dots presented on a computer monitor in the absence of depth cues create a bistable percept of a rotating sphere (see Supplemental Experimental Procedures for details). The percept for this structure from motion typically alternates between the two possible revolution directions. (B) Fifty-two participants reported their percepts. The frequency histogram shows the number of participants as a function of the average percept duration that they reported using a 1 s bin width. See Results for full details.

We recorded subjective reports of spontaneous alternations for an ambiguous rotating structure-from-motion (SFM) stimulus that evokes bistable perception fluctuating between two rotation directions (Figure 1A) [10]. Across the group (n = 52), there was substantial variability in the mean duration of each percept and its reciprocal, the switch rate (Figure 1B). We tested whether this variability in perception was predicted by variability in brain structure by using three measures: gray matter (GM) density and cortical thickness measured via anatomical magnetic resonance imaging scanning, and the fractional anisotropy (FA) of white matter (WM) measured using diffusion tensor imaging. Potentially confounding factors of gender and age, which affect brain structure [11], were regressed out and thus removed as part of this analysis (see Experimental Procedures).

### Cortical Thickness

**Figure 2:**
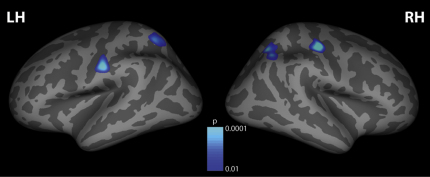
Results of Cortical Thickness Analyses Shown in blue are those cortical loci where our analyses of cortical thickness revealed a significant negative correlation between cortical thickness and percept duration (p < 0.05, corrected) across the group of participants. Data are shown overlaid onto an inflated template brain in a standard stereotactic space where sulci are represented in dark gray and gyri in light gray. The significant clusters are shown at a threshold of p < 0.01 for visualization purposes; see Table S1 for statistical values and stereotactic location of activated foci.

We found a significant negative correlation between cortical thickness and percept duration across individuals in the bilateral superior parietal lobule (left p < 0.05, right p < 0.01, corrected for multiple comparisons) and bilateral postcentral gyrus (left p < 0.05, right p < 0.01, corrected for multiple comparisons) (Figure 2). The negative correlation with percept duration indicated that the thicker the cortex in these regions, the faster the switch rate of an individual. No other loci, including prefrontal regions that are activated by perceptual switches [12–14], showed any significant correlation with percept duration (all p > 0.05). The details of the results are summarized in Table S1 available online.

### Voxel-Based Morphometry

**Figure 3:**
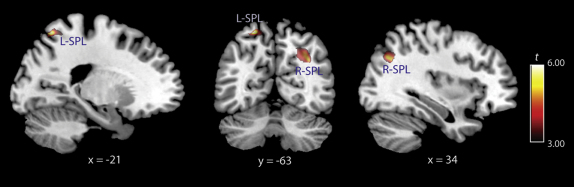
Results of GM Volume Voxel-Based Morphometry Analysis Shown in red are the left and right superior parietal lobe (SPL) loci that showed significant negative correlations between GM density and percept duration superimposed on a standard template created in a standard stereotactic space from all of the participants (see Supplemental Experimental Procedures for full details). A threshold of T > 3.00 was used for visualization purposes; see Table S2 for statistical values and stereotactic location of activated foci.

To cross-validate our findings, we conducted a voxel-based morphometry (VBM) analysis of GM density [15] on the same data set (see [16] for discussion of similarities and differences in cortical thickness and VBM analyses of gray matter). Consistent with the cortical thickness analysis, we found significant correlations between GM density and an individual's percept duration across the group in the right superior parietal lobe (SPL) (x = 34, y = −66, z = 34, p < 0.05, corrected) and left SPL (x = −21, y = −63, z = 61, p < 0.01, corrected) (Figure 3). However, we did not find a correlation in the postcentral gyri even with a liberal statistical threshold (p > 0.05, uncorrected).

The differences between cortical thickness and GM density analyses could be due to a number of factors such as measurement of different aspects of GM structure [16]. On the other hand, our consistent findings between the two different measures show a robust association between the bilateral SPL loci and perceptual alternations. Again, the VBM analysis did not reveal significant correlations between GM density and switch rate in any other region of the brain (p > 0.05, corrected). The details of the results are summarized in Table S2.

### Diffusion Tensor Imaging

Having established a significant correlation between an individual's bistable percept duration and GM thickness/density in the bilateral parietal cortex, we next examined the contribution of WM integrity to individual differences in the bistable percept duration. This analysis used an independent data set of diffusion-weighted images (DWIs). The FA values derived from DWIs provide an estimate of the integrity of WM tracts [17]. To investigate whether this indirect measure of WM connectivity was associated with individual differences in percept duration, we determined whether there were significant correlations between FA and an individual's bistable percept duration. Importantly, our earlier results described above (i.e., right SPL and left SPL) that were obtained from the analyses of the gray matter now served as strong prior hypotheses for where in the brain the strongest correlations with WM connectivity should be found.

**Figure 4:**
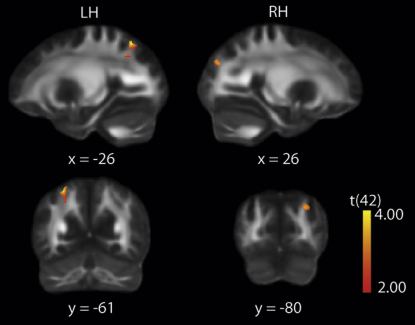
Fractional Anisotropy Analysis Shown in red are the left and right SPL loci that showed significant negative correlations between fractional anisotropy (FA) and percept duration, superimposed on the standard FMRIB58_FA (http://www.fmrib.ox.ac.uk/fsl/data/FMRIB58_FA.html) FA image in a standard stereotactic space. A threshold of T > 2.00 was used for visualization purposes. See Table S3 for details of loci depicted.

The results of this analysis are shown in Figure 4. Consistent with the cortical thickness and VBM analyses described above, we found significant negative correlations between FA values and bistable percept duration in the white matter beneath the left SPL (x = −26, y = −61, z = 50; p < 0.05, small volume correction [SVC] corrected) and the right SPL (x = 26, y = −80, z = 31; p < 0.05 SVC corrected). These locations were highly consistent with the coordinates for those regions found with the cortical thickness and the VBM analyses, although of course slightly displaced, reflecting the origin of the FA signal in white (not gray) matter.

### Transcranial Magnetic Stimulation

Our findings so far only establish that brain structure is correlated with fluctuations in conscious perception and cannot on their own determine whether these structural variations also play a causal role in generating such perceptual switches. In addition, our finding of variability in homologous bilateral cortical structures could conceivably reflect unrelated covariation between hemispheric structures. For example, GM density in parietal regions correlates with the homotopic region in the contralateral hemisphere [18]. Therefore, it is possible that even if only one of the regions revealed by the correlation analyses were causally related to the individual differences in perception, other contralateral regions around the homotopic locus might show a statistically significant correlation as a result of their coordinated variations with the critical area.

Therefore, we now investigated whether the anatomical loci that we identified also played a causal role in mediating switches by using transcranial magnetic stimulation (TMS). In separate sessions, we temporarily disrupted the function of right SPL or left SPL (using the group coordinates revealed by our interindividual structural variability experiments above) by delivering continuous theta-burst stimulation (cTBS) [19]. If activity in these parietal loci had any direct causal influence on an individual's switch rate, we hypothesized that the application of cTBS to each area should change the switch rate compared to application of TMS to a control site (vertex).

**Figure 5:**
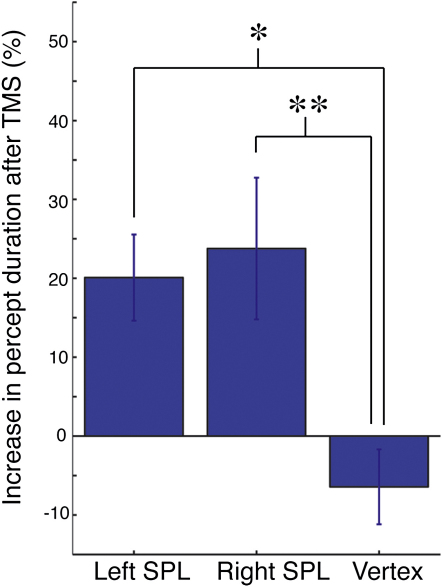
Effects of Theta-Burst Stimulation on Bistable Percept Duration The mean changes in percept duration, computed as percentage change relative to the baseline percept duration before theta-burst stimulation, are plotted for left SPL, right SPL, and vertex conditions. ^∗^p < 0.05, ^∗∗^p < 0.01 between the indicated conditions by post hoc least significant difference test. Error bars correspond to one standard error of the mean across ten participants.

Repeated-measures analysis of variance on the changes in percept duration with respect to cTBS revealed that there was a significant difference among the stimulation conditions (F(1,9) = 15.2, p < 0.01). Post hoc least significant difference tests revealed that cTBS over the right SPL and the left SPL significantly increased percept durations compared to the control condition (right SPL p < 0.01, left SPL p < 0.05) (Figure 5). These results show that the areas where we found that structural variability could account for individual differences in conscious perception also play a causal role in determining perceptual switch rate.

## Discussion

Taken together, our findings reveal that differences in brain structure in focal regions of bilateral parietal cortex can account for individual differences in conscious visual perception, and that the same regions showing such structural variability also play a causal role in such perception. Between-participant variability in bistable perception has been documented, but the neural basis underlying the variability has been neglected for many years [2–5]. The results presented here thus provide for the first time a neurobiological basis for the individual differences in perceptual bistability.

The SPL loci identified here are anatomically very close to those parietal regions that show transient activations when participants shift their attention between locations [20, 21] or between spatially overlapping objects [22]. They also coincide with very similar regions that are activated at the time of perceptual switches in various types of perceptual rivalry [12, 13]. Our findings suggest that individual differences in switch rate arise from individual differences in the GM density within those areas that may therefore be related to attention (and percept) switching. They therefore provide a potential link between changes in brain structure and the functional role of such regions that could be explored in future work.

Our findings offer a possible neural account for hitherto unexplained relationships that have been found between switch rate and the effects of psychiatric mood disorders, normal aging, or brain damage. For example, patients with bipolar disorder show cortical thinning compared with matched controls in multiple cortical regions, including a region (x = 38, y = −64, z = 32) [23] close to the right SPL region that we identified here where a correlation between GM density, cortical thickness, and switch rate is shown. Moreover, we found that thinner cortex in this area was associated with slower switch rate, consistent with the thinner SPL cortex [23] and slower rivalry in bipolar patients [24]. In addition, the switch rate of ambiguous stimuli decreases with age [25]. This might also be attributed to GM shrinkage with aging, because GM volume reduction with aging is particularly severe in the parietal regions [26]. Our findings that parietal regions are causally involved in spontaneous perceptual alternations also accounts for abnormally slow perceptual rivalry following damage to parietal cortex [27]. Thus, our findings provide a potentially unifying explanation for a number of so far apparently unconnected phenomena.

A recent study that compared monozygotic and dizygotic twins found that about half (52%) of the variability in spontaneous switches in perceptual rivalry can be accounted for by genetic factors [28]. Our present findings suggest that such genetic influence on individual differences in perceptual switch rate might be mediated by their impact on the development and maturation of the SPL.

How differences in the structure of SPL result in differences in perceptual switch rate remains unclear. One possible mechanism is the differences in the strength of feedback signals from SPL to early sensory areas that reset the neuronal activities supporting the current percept. If the SPL is large and strongly connected to early visual areas, the impact of the feedback signals would also be stronger and therefore trigger perceptual switches at a higher rate.

In the present study, we used SFM as a representative bistable stimulus. Thus, whether our findings generalize to a broader range of bistable stimuli remains an open question. However, previous studies suggest the existence of neural substrates common to a range of bistable stimuli. For example, temporal pattern of alternations reported during binocular rivalry is highly correlated with that of motion-induced blindness [5]. Moreover, strong correlation has been reported between perceptual switch rates for auditory rivalry and visual plaid rivalry [29], suggesting common mechanisms even across different modalities. On the other hand, there is evidence that binocular rivalry, compared to more general perceptual rivalry, might be mediated by low-level sensory processing (e.g., [30, 31]). Further studies are warranted to determine whether the structure of SPL predicts switch rates for other types of rivalry stimuli such as binocular rivalry or auditory rivalry.

Previous functional neuroimaging [12–14] and stroke patient [32, 33] studies showed possible involvements of the prefrontal cortex in spontaneous alternations. However, our present study did not find any prefrontal region that correlated with individuals' switch rate. One possibility is that even though prefrontal regions did correlate with spontaneous switch rate, the correlation was weaker and did not survive our stringent statistical criterion (i.e., whole-brain family-wise error correction). To examine this possibility, we specifically examined the previously reported prefrontal loci using small-volume correction. However, none of the previously reported regions, including the frontal loci, showed a significant correlation with individuals' switch rate, even with a more liberal statistical threshold (see Supplemental Results). This suggests that even though prefrontal regions are activated at the time of spontaneous alternations, their structure does not directly predict individuals' switch rate.

Finally, our work shows that intervention with TMS may be a particularly powerful approach for validating findings from independent structural analyses of the brain. Our approach of linked anatomical and functional studies may be particularly relevant and useful for addressing a situation that may arise in large-scale correlational studies such as VBM. Highly specific interventional studies with TMS can be combined with highly sensitive correlational methods such as VBM and FA to confirm the causal link between the behavior of interest and brain regions identified by structural analyses.

Taken together, our findings reveal that in humans, the cortical thickness and GM density in remarkably focal regions of parietal cortex can account for differences in how conscious visual perception fluctuates over time, and that the same regions showing this structural variability also play a causal role in bistable perception. We speculate that other aspects of conscious visual perception may similarly have an unexpected motif in human brain structure.

## Experimental Procedures

Full details of the methods used are provided in the Supplemental Experimental Procedures.

### Participants

A total of 52 healthy volunteers were recruited for the individual differences experiment; 12 healthy volunteers were recruited for the TMS experiment. We obtained written informed consent from all participants, and the experiments were approved by the local ethics committee (University College London).

### Individual Differences Experiment

#### Cortical Thickness

Reconstruction of the pial surface and GM/WM boundary was performed for T1-weighted magnetic resonance (MR) images via the fully automated procedure implemented in FreeSurfer software [34, 35]. The thickness measures were smoothed with a Gaussian kernel over the extracted surfaces (full width half maximum [FWHM] = 15 mm). A multiple regression was performed to identify cortical regions that show a correlation with individuals' percept duration. The gender and age of each participant was included in the multiple regression analysis design matrix as covariates of no interest to model and thus regress out any effects attributable to age or gender [11, 26]. Cluster-wise correction for multiple comparisons was performed by Monte Carlo simulation with 10,000 simulations for estimating the probability of forming a cluster of a given size by chance when clusters were obtained by a vertex-wise threshold of p < 0.005. We took a threshold of cluster-wise p values of 0.05 as the criterion for significant clusters.

#### Voxel-Based Morphometry

The same set of T1-weighted MR images were used for the VBM analysis. The MR images were first segmented for gray matter and white matter by using the segmentation tools in SPM8 (http://www.fil.ion.ucl.ac.uk/spm). Subsequently, we performed diffeomorphic anatomical registration through exponentiated lie algebra (DARTEL) [36] for intersubject registration of the GM images. The registered images were smoothed with a Gaussian kernel (FWHM = 8 mm) and then transformed to Montreal Neurological Institute stereotactic space for multiple regression analysis. The gender and age of the participants were included in the design matrix as covariates of no interest. We used p < 0.05 family-wise error corrected for the whole-brain volume as the criterion to detect voxels with a significant correlation with an individual's percept duration.

#### Fractional Anisotropy

FA was calculated by using Functional Magnetic Resonance Imaging of the Brain (FMRIB)'s Diffusion Toolbox (FDT v2.0) applied to the diffusion-weighted MR images. FA images were coregistered with a standard template (FMRIB58). The standardized FA images were smoothed with an isotropic Gaussian kernel (FWHM = 8 mm) for multiple regression analysis. We used SVC based on the coordinates of parietal sites revealed in the VBM analysis. We used p < 0.05 corrected for the small volume as the criterion to detect voxels with a significant correlation with an individual's percept duration.

### TMS Experiment

The stimuli and procedure for the TMS experiment were identical to the experiment used for estimating individuals' percept duration for the correlation studies above. Mean percept duration was compared before and after cTBS. Participants completed three blocks reporting their ambiguous motion percepts before the TMS session and another three blocks immediately after. The three stimulation sites (right SPL, left SPL, and vertex) were tested on separate days, and the order of the sites was randomized for each subject.

## Supplemental Information

Document S1. Four Tables, Supplemental Results, Supplemental Discussion, and Supplemental Experimental Procedures
