# Supplementary material for: Human Parietal Cortex Structure Predicts Individual Differences in Perceptual Rivalry
Source: Curr Biol. 2010 Sep 28;20(18-4):1626–30. doi: 10.1016/j.cub.2010.07.027 (PMC2949566; doi:10.1016/j.cub.2010.07.027)
Supplement: Document S1. Four Tables, Supplemental Results, Supplemental Discussion, and Supplemental Experimental Procedures [file mmc1.pdf]

Current Biology, Volume *20*

Supplemental Information

Human Parietal Cortex Structure Predicts Individual Differences in Perceptual Rivalry

Ryota Kanai, Bahador Bahrami, and Geraint Rees

Table S1.

		MNI Coordinates (mm)			T	p corrected
**Region**	**Hemi**	**x**	**y**	**z**		
Superior Parietal lobe	R	28.7	-54.8	45.1	3.98	<0.01
	L	-28.3	-51.8	54.1	3.40	< 0.05
Postcentral gyrus	R	42.5	-16.8	46.5	5.17	< 0.01
	L	-59.0	-9.8	33.4	4.30	< 0.01

The MNI coordinates and statistical results are summarised for the peak vertices of the clusters found to correlate significantly with percept duration ($p < 0.05$, corrected for multiple comparison).

Table S2.

		MNI Coordinates (mm)			T	Z	p (FWE corrected)
**Region**	**Hemi**	**x**	**y**	**z**			
Superior parietal lobe	R	34	-66	34	5.27	4.65	p < 0.05
	L	-21	-63	61	5.98	5.13	p < 0.01

The MNI coordinates and statistical results are summarised for the peak voxels that showed significant correlation with percept duration ($p < 0.05$, FWE-corrected for multiple comparison). No other regions including postcentral gyri showed significant correlation ($p > 0.05$, corrected).

Table S3.

		MNI Coordinates (mm)			T	Z	p (s.v.c.)
**Region**	**Hemi**	**x**	**y**	**z**			
Superior parietal lobe	R	27	-78	30	3.97	3.65	p < 0.05
	L	-28	-61	51	4.18	3.82	p < 0.01

The MNI coordinates and statistical results for FA analysis are summarized. The peak voxels that showed significant correlation with percept duration ($p < 0.05$, corrected for small volume defined by a spheres centered at the peak coordinates of VBM results).

Table S4.

		Coordinates		
**Area**	**Hemisphere**	**x**	**y**	**z**
Occipital	RH	3	-99	6
Fusiform	LH	-33	-54	-21
	RH	36<br>42	-42<br>-51	-27<br>-27
SPL	LH	-18	-57	45
	RH	21	-60	48
IFG	LH	-57	12	9
	RH	48	24	9
LPFC	LH	-30	0	42
	RH	36	0	45
PCC	LH	-3	-42	27
Thalamus	LH	-9	-30	3
	RH	9	-30	3

The MNI coordinates used for small volume correction analysis described in Supplemental Results are listed (S10). None of the spheres (15 mm radius) centred at these coordinates except for the bilateral SPLs contained a voxel with significant correlation ($p < 0.05$, uncorrected).

Supplemental Results

For regions known to exhibit transient activation (or deactivation) to spontaneous perceptual switches in bistable stimuli (S9-S11) we used the small volume correction at the MNI coordinates of the loci reported in ref. S10 (see, Table. S4). The regions from ref. S10 were used because the bistable stimuli used in that study were reversible figures and were more compatible with the SFM stimuli we used. Also, the loci reported in ref. S10 covers the areas reported in other studies (S9, S11).

For each of these regions, we defined a sphere centred at the reported MNI coordinate and examined if they contain a voxel with significant correlation with percept duration using an extremely lenient statistical threshold ($p < 0.05$, uncorrected) using the same data set used for the VBM analysis. However, none of the spheres except for the bilateral SPL contained a significant voxel. This indicates that there is not even weak correlation between grey matter density and individual's switch rate in the regions previously implicated in spontaneous perceptual switch. These results suggest that SPL is the only area that directly predicts individual perceptual switch rate.

Supplemental Discussion

Relationship between GM Density and WM Integrity

All the analyses of GM thickness/density and WM integrity consistently revealed that cortical thickness, GM density and underlying WM integrity of left SPL and right SPL cortices were significantly correlated with an individual's percept duration. To further examine the relationship between local GM density and FA values, we computed the correlation between these two measures after factoring out age and gender contributions to these effects in the left SPL and right SPL foci.

There was a significant correlation across the group of participants between local GM density and FA values (left SPL, $R = 0.458$, $p < 0.01$; right SPL, $R = 0.378$, $p < 0.01$). Thus differences in GM density were also reflected in WM integrity. This is in line with the finding that training to juggle (i.e. learning a complex visuomotor skill) increases the FA values associated with WM tracts co-localized with medial occipital and parietal regions that also show training-associated GM volume increases (S8). However they go beyond such data by now showing that GM and WM integrity can reflect spontaneous, innate and untrained aspects of conscious sensory perception.

Supplemental Experimental Procedures

Participants

For the GM MRI analyses (cortical thickness and VBM), a total of 52 healthy volunteers with normal or corrected to normal vision (ages 19–38, mean 25.8 ± 5.14 S.D.; 29 female) were recruited. For the WM MRI analysis (fractional anisotropy), a total of 46 healthy volunteers (ages 19–38, mean 26.2 ± 5.27 S.D.; 28 female) were recruited. The participants in the FA analysis were a subpopulation of the 52 participants in the cortical thickness and VBM analyses. We obtained written informed consent from all participants before the behavioral task and the scanning sessions. The local ethics committee approved the experiments.

Behavioral Task

For the individual differences experiment, an ambiguous rotating sphere comprising 200 full-contrast white dots and subtending 3.5º diameter was presented against a black background on a 17-inch monitor using PsychToolbox (S1, S2) running under MATLAB (The Mathworks, Inc.). The dots moved sinusoidally with an angular velocity of 151 deg/s. A red fixation cross (0.5º in height and width) was superimposed at the centre of the sphere to aid steady fixation. On each trial, the dots comprising the ambiguous rotating sphere were presented continuously for 48 seconds. Participants were instructed to report the perceived direction of the rotation of the sphere by holding down one of two keys. They were also instructed not to press any key when the percept was unclear. Before the main experiment, participants were given practice trials to ensure they understood the task and instructions. Then, they completed 8 runs of trials (a total of 384s) for data acquisition to be used in subsequent correlation analyses.

MRI Data Acquisition

MR images were acquired on a 1.5-T Siemens Sonata MRI scanner (Siemens Medical, Erlangen, Germany). High-resolution anatomical images were acquired using a T1-weighted 3-D Modified Driven Equilibrium Fourier Transform (MDEFT) sequence (TR = 12.24 ms; TE = 3.56 ms; field of view = 256 x 256 mm; voxel size = 1 x 1 x 1 mm). The diffusion-weighted images (DWIs) were measured in 68 non-collinear directions (TR = 160ms; TE = 90ms; flip angle = 90º; field of view =; matrix size = 96 x 96; 60 slices; b = 1000, 100ms).

Data Analyses

Cortical Thickness: Reconstruction of the pial surface and GM/WM boundary was performed for 52 T1-weighted MR images using the fully automated procedure implemented in Freesurfer software (S3). The thickness was computed as the shortest distance between the GM/WM boundary and the pial surface (S4) and the thickness measures were transformed using the surface-based co-registration of each individual's folding patterns to a standard template. The thickness measures were smoothed with a Gaussian kernel over the extracted surfaces (FWHM = 15mm). A multiple regression was performed to identify cortical regions that show a correlation with individual's percept duration. The gender and age of each participant was included in the multiple regression analysis design matrix as covariates of no interest to model and thus regress out any effects attributable to age or gender (S5). This treatment removes potential confounds of these factors. Cluster-wise correction for multiple comparisons was performed by Monte Carlo simulation with ten thousands simulations for estimating the probability of forming a cluster of a given size by chance when clusters were obtained by a vertex-wise threshold of $p < 0.005$. We took a threshold of clusterwise p-values of 0.05 as the criterion for significant clusters.

Voxel-Based Morphometry: The same set of T1-weighted MR images as in the cortical thickness analysis were used for the VBM analysis. The MR images were first segmented for GM and WM using the segmentation tools in SPM8 (http://www.fil.ion.ucl.ac.uk/spm). Subsequently, we performed Diffeomorphic Anatomical Registration Through Exponentiated Lie Algebra (DARTEL) (S6) in SPM8 for inter-subject registration of the GM images. The registered images were smoothed with a Gaussian kernel (FWHM = 8 mm) and were then transformed to MNI stereotactic space using affine and non-linear spatial normalisation implemented in SPM8 for multiple regression analysis. The gender and age of the participants were included in the design matrix as covariates of no interest to model and thus regress out any effects correlated with these factors. We used $p < 0.05$ family-wise error corrected for the whole brain volume as the criterion to detect voxels with a significant correlation with individual's percept duration.

Fractional Anisotropy: Fractional anisotropy (FA) was calculated using FMRIB's Diffusion Toolbox (FDT v2.0) applied to the diffusion-weighted MR images. FA images were coregistered with a standard template (FMRIB58) using FSL's non-linear image registration tool (FNIRT). The standardized FA images were smoothed with an isotropic Gaussian kernel (FWHM = 8 mm) for statistical analysis. We used small volume correction (s.v.c.) based on the coordinates of parietal sites revealed in the VBM analysis. We used $p < 0.05$ corrected for the small volume as the criterion to detect voxels with a significant correlation with an individual's percept duration. Outside those regions we used a threshold of $P<0.05$, corrected for multiple comparisons across the whole brain volume.

TMS Experiment

Stimuli and Procedure: Ten healthy participants with normal or corrected-to-normal vision gave written informed consent to participate in three TMS sessions, in a protocol that was approved by the local ethics committee. The stimuli and procedure were identical to the experiment used for estimating individuals' percept duration for the correlation studies above, except that theta-burst TMS stimulation was applied between two of the blocks (see below for details). Participants completed 3 blocks reporting their ambiguous motion percepts before the TMS session and another 3 blocks immediately after. The mean percept duration for pre-TMS was calculated based on the second and third blocks. The mean percept duration for post-TMS was calculated from all the 3 blocks. The three stimulation sites (right SPL, left SPL and vertex) were tested on separate days and the order of the sites was randomized for each subject.

TMS Protocol: TMS pulses were delivered by a Magstim Rapid Stimulator at 45% of stimulator output using a 70-mmm figure-of-eight coil. The position of left SPL and right SPL were obtained by converting the coordinates obtained from the GM volume analysis for individual participants using FSL software (FMRIB, Oxford). The target sites determined for individual participants were used to guide TMS coil position using a frameless stereotaxic system (Brainsight, Rogue Research, Canada). We delivered the standard continuous theta-burst stimulation (cTBS) protocol, i.e., 3 pulses at 50Hz repeated at 200 ms intervals (S7) for 40 seconds. Such a protocol is known to depress cortical function below the stimulated region for between 10 and 20 minutes.

Supplemental References

S1. Brainard, D.H. (1997). The Psychophysics Toolbox. *Spat. Vis.* 10, 433-436.

S2. Pelli, D.G. (1997). The VideoToolbox software for visual psychophysics: transforming numbers into movies. Spat. Vis. 10, 437-442.

S3. Fischl, B., Sereno, M.I. and Dale, A.M. (1999). Cortical surface-based analysis. II: Inflation, flattening and a surface-based coordinate system. Neuroimage *9*, 195-207.

S4. Fischl, B. and Dale, A.M. (2000). Measuring the thickness of the human cerebral cortex from magnetic resonance images. Proc. Natl. Acad. Sci. USA *97*, 11050-11055.

S5. Smith, C.D., Chebrolu, H., Wekstein, D.R., Schmitt, F.A. and Markesbery, W.R. (2007). Age and gender effects on human brain anatomy: A voxel-based morphometry study in healthy elderly. Neurobiol. Aging, *28*, 1075-1087.

S6. Ashburner, J. (2007). A fast diffeomorphic image registration algorithm. *NeuroImage* 38, 95-113.

S7. Huang, Y.-Z., Edwards, M.J., Rounis, E., Bhatia, K.P. and Rothwell, J.C. (2005). Theta burst stimulation of the human motor cortex. Neuron *45*, 201-206.

S8. Scholz, J., Klein, M.C., Behrens, T.E.J. and Johansen-Berg, H. (2009). Training induces changes in white-matter architecture. Nat. Neurosci. *12*, 1370-1371.

S9. Lumer, E.D., Friston, K.J. and Rees, G. (1998). Neural correlates of perceptual rivalry in the human brain. Science *280*, 1930-1934.

S10. Kleinschmidt, A., Büchel, C., Zeki, S. and Frackowiak, R.S. (1998). Human brain activity during spontaneously reversing perception of ambiguous figures. Proc. R. Soc. Lond. B *265*, 2427-2433.

S11. Sterzer, P. and Kleinschmidt, A. (2006). A neural basis for inference in perceptual ambiguity. Proc. Natl. Acad. Sci. U.S.A. *104*, 323-328.
